# Redox-sensitive miRNAs and Humanin could mediate effects of exercise and astaxanthin on oxidative stress and inflammation in type 2 diabetes

**DOI:** 10.1038/s41598-025-23914-y

**Published:** 2025-11-17

**Authors:** Aref Basereh, Karen Khoramipour, Najmeh Hosseini, Mahdieh HajHosseini, Adeleh Khodabakhshi, Ladan Amirkhosravi, Kayvan Khoramipour

**Affiliations:** 1 Department Exercise Physiology, Kharazmi University, Tehran, Iran; 2 Faculty of Humanities and Social Sciences, Department of Sport Science, Kurdistan University, Kurdistan, Iran; 3 Physiology and Neuroscience Research Center, Institute of Physiology and Pharmacology, Kerman University of Medical Sciences, Kerman, Iran; 4 Department of Physical Education, Faculty of Humanities and Arts, , National University of Skills (NS), Tehran, Iran; 5 Department of Nutrition, Faculty of Public Health, Kerman University of Medical Sciences, Kerman, Iran; 6 Endocrinology and Metabolism Research Center, Kerman University of Medical Sciences, Kerman, Iran; 7 I+HeALTH Strategic Research Group, Department of Health Sciences, Miguel de Cervantes European University (UEMC), 47012 Valladolid, Spain

**Keywords:** miRNAs, Astaxanthin supplementation, Humanin, T2DM, Combined training

## Abstract

Type 2 diabetes mellitus (T2DM) is driven by oxidative stress (OS) and inflammation (IF), accelerating disease progression. This study examined whether combined aerobic and resistance training (CT) and astaxanthin (AST) supplementation synergistically improve oxidant and inflammatory status as well as metabolic indices in T2DM, focusing on the mediatory role of Humanin (HN) and microRNAs (miRNA-122, miRNA-126-3p, and miRNA-146a). Ninety women with T2DM were randomly assigned to six groups (n = 15 each): control (C), placebo (P), AST supplementation (S), combined training (CT), CT + placebo (CT + P), and CT + AST supplementation (CT + S). CT, CT + P and CT + S groups underwent an 8-week training program (eight exercises, three sessions per week). Groups and CT + S groups received 8 mg/day of AST. OS markers, inflammatory cytokines, HN levels, miRNAs expression, fasting blood glucose (FBG), insulin resistance (HOMA-IR), lipid profile, and hemoglobin A1c (HbA1c) were assessed. Both CT and AST enhanced antioxidant defenses and reduced IF, with CT + S showing the best effects. HN levels increased significantly in CT and CT + S (*p* < 0.05). MiRNA-126-3p and miRNA-146a were upregulated, while miRNA-122 was downregulated in CT + S compared to other groups. Lipid profile improved with both interventions, with CT + S yielding the highest increases in HDL and triglycerides. FBG, IR, and HbA1c improved significantly in CT groups but remained unchanged with S group. The metabolic and anti-inflammatory benefits of CT and AST in T2DM may mediated by the effects of HN on mitochondrial function and insulin signaling, together with miRNA-mediated regulation of lipid metabolism, endothelial health, and innate immunity. Targeting these molecular pathways may improve therapeutic strategies for diabetes management.

**Supplementary Information:**

The online version contains supplementary material available at 10.1038/s41598-025-23914-y.

## Introduction

The number of adults living with type 2 diabetes mellitus (T2DM) has increase rapidly over the years. In 2000, around 150 million people were affected, but by 2019, that number had more than tripled to over 450 million. If current trends continue, this figure will rise to about 700 million by 2045. T2DM develops through a combination of factors, including high blood sugar levels, unhealthy fat levels in the blood, oxidative stress (OS), and inflammation (IF)^1^. High blood sugar can lead to the production of harmful free radicals, which can damage lipids, proteins, and even our DNA. Additionally, when pancreatic beta cells are exposed to oxidative stress, it can hinder their ability to produce insulin by affecting the genes responsible for insulin production^2^^,^^3^. Furthermore, hyperglycemia-induced OS is thought to elevate the levels of pro-inflammatory proteins and cytokines^4^. Chronic IF in insulin-responsive tissues is a key contributor to insulin resistance (IR), and this condition is often linked to elevated levels of reactive oxygen species (ROS)^5^. The interaction of IF and OS results in a worsened vicious cycle that impairs insulin signaling and gives rise to IR^1^.

It has been proposed that the most optimal way to manage OS and IF is through a healthy lifestyle^5,6^. Two fundamental parts of a healthy lifestyle include being physically active and having a proper diet. Regular exercise is associated with increased release of anti-inflammatory cytokines and lower concentrations of some pro-inflammatory cytokines^7^. According to Sabouri et al. study^8^ the addition of high-intensity interval training (HIIT) to strength training programs significantly improved the patient’s IF, antioxidant defense, and glycemic control to the previously existing conditions of the patient. On the other hand, resistance training has gained attention for its benefits in augmenting muscle mass and strength and controlling glucose levels through enhanced insulin sensitivity in the skeletal muscle^9,10^ both of which are important in the management of T2DM. Combined aerobic and resistance training (CT) has emerged as combination training due to its multi-faceted benefits which enhance glucose metabolism, IF, and OS^12,13^. Aerobics and resistance training, when done together, provide synergistic advantages which are unique to both forms of training. While CT enhances aerobic fitness, it also enables muscle hypertrophy that improves insulin sensitivity and body composition^14^. Additionally, 8 weeks of combined training also enhanced physical fitness and improved body composition as well as IF^15^.

Healthy diet and supplementation are the other frequently used methods in reducing T2DM symptoms^16^. Astaxanthin (AST) is a xanthophyll carotenoid with antioxidant and anti-inflammatory properties, which, unlike those of other carotenoids, can integrate into membrane and scavenge free radicals thanks to its unique molecular structure^17^. Clinical trials showed that AST has the ability to lower oxidative stress, IF, and promote the function of antioxidant enzymes like superoxide dismutase (SOD) and catalase^17^. AST can also improves insulin sensitivity, PI3K/Akt pathways, and also protects the mitochondria, which are relevant in the pathophysiology of T2DM^18,19^. These aspects make AST a promising therapy for the treatment of diabetes and diabetes-related metabolic disorders, especially when combined with lifestyle changes such as structured exercise training.

Previous studies have focused on classical signaling pathways that can explain anti-oxidant and anti-inflammatory effects of exercise and AST. However, there are newly introduced signaling pathways and molecules that can play important role in this process^20,21^. Investigating these novel molecules and their roles in mitigating oxidative stress and inflammation may open promising avenues for both research and therapeutic development. Humanin (HN) is one such recently recognized molecule with a crucial role in reducing OS and IF^22–24^. HN is a member of a class of novel mitochondrial-derived peptides released during mitochondrial dysfunction^25^. HN reduces ROS production, enhances antioxidant protein expression, maintains redox balance^24^, and suppresses TNF-α, IL-1β, and IL-6 to inhibit IF^23^. Furthermore, resistance and endurance training has shown to increases HN expression in patients with prediabetes^16^. Exercise—aerobic and endurance- has been shown to increase circulating and skeletal muscle levels of HN, correlating with improved insulin sensitivity and mitochondrial function^26,27^. HIIT is known to have beneficial effects for T2D rats by reducing both IF and OS. Considering what is already known about HN’s role in these processes, it seems probable that the benefits of HIIT for T2D rats are due, at least in part, to HN^28^.

Another newly discovered molecules with bold role in improving antioxidant and anti-inflammatory response are microRNAs (miRNAs)^29^. miRNA-122, miRNA-126-3p and miRNA-146a showed significant association with inflammatory cytokines as well as IR in diabetes and pre-diabetes patients^30^. MiRNAs also play a crucial role in regulating the molecular mechanisms responsible for β cell dysfunction and their response to OS^31^. MiRNA-146a acts as a negative regulator of inflammation by targeting key pro-inflammatory genes involved in the nuclear factor kappa light chain enhancer of activated B cells (NF-κB) pathway^32^. MiR-146a inhibits overproduction of ROS proinflammatory cytokines^33^. AST supplementation decreased circulating levels of miR-146a and was associated with reduced IL-6 and MDA in T2DM^22^. Two independent reports have shown that aerobic training can increase miR-146a levels^24,25^, which may help manage the chronic inflammation often seen in T2DM^25^.

MiRNA-122 is specifically expressed in the liver where it is believed to modulate the inflammatory response and insulin resistance in T2D^30^. It is known that increased levels of miRNA-122 are linked to increase IF and OS, which worsens metabolic dysfunction in diabetes^34^. Combined exercise could decrease miRNA-122 expression and, in turn, may improve the components of metabolic syndrome^35^. Downregulation of miR-122 could inhibit OS through PI3K/AKT signaling pathway^34^.

Endothelial cells express miRNA-126-3p, and it is important for maintaining vascular health as well as insulin signaling and angiogenesis^36^. Aerobic training^37,38^ shown to increase levels of miRNA-126-3p, which improves vascular function and increases insulin sensitivity. Given that insulin signaling and angiogenesis are critical for metabolic balance, the further increase in miRNA-126-3p levels in response to exercise and AST supplementation may help explain the observed therapeutic impact in T2D.

Since both exercise and AST can potentially impact HN and certain microRNAs that regulate OS and IF, our study aimed to explore how the combination of CT and AST affects these processes. Specifically, we looked at how they influence HN, miRNA-122, miRNA-126-3p, and miRNA-146a, with the ultimate goal of reducing the symptoms and complications associated with diabetes.

## Material and methods

### Experimental procedure

This study was a double-blind, randomized, placebo-controlled trial and 90 volunteer’s women with T2DM were enrolled in it. Kurdistan University of Medical Sciences referred the participants and they were selected based on specific inclusion criteria: age between 30 and 60 years old, had a confirmed diagnosis of T2DM with no insulin usage, were not pregnant or breastfeeding, and did not have chronic diseases, cancers, kidney failure, heart disease, thyroid disorders, or other inflammatory conditions. Additionally, the participants had not used vitamins or antioxidant supplements in the previous 6 months and were non-smokers and non-alcohol consumers. Informed written consent was obtained from each participant before commencing the study procedures. Ethics Committee of Kerman University of Medical Sciences approved all procedure (Approval Code: IR.KMU.REC.1401.530). This study was registered in the Iranian Registry of Clinical Trials (IRCT20230225057524N1) on 20/03/2023. The research was conducted in full compliance with the Declaration of Helsinki, Iranian national research ethics guidelines, and the International Committee of Medical Journal Editors (ICMJE) recommendations for clinical trials.

Participants were randomly assigned to one of the six groups (n = 15 in each): Control (C), placebo (P), AST supplementation (S), Combined training (CT), CT + placebo (CT + P), and CT + AST supplementation (CT + S). The exercise sessions were conducted for 8 weeks, with 3 sessions per week. Before starting the exercise programs, all participants were familiarized with the environment and exercises. Before the study began and 72 h after the final training or supplementation session, we measured the participants’ body composition, height, and weight. Each participant also went through initial evaluations by a physical medicine specialist and an endocrinologist. We excluded individuals from the study if they had any of the following conditions: a history of ketoacidosis within the past six months, high blood sugar levels (HbA1c > 10%), diabetic complications affecting small blood vessels, diabetic neuropathy, liver issues (indicated by elevated plasma aminotransferase or gamma-glutamyl transferase levels for their age and gender), kidney problems (with serum creatinine levels above normal for their age and gender), severe anemia, uncontrolled high blood pressure, cardiovascular diseases (such as congestive heart failure or a history of heart attacks), or any cerebrovascular incidents.

### Exercise protocol

CT, CT + P, and CT + S groups performed the following exercises three time a week for 8 weeks:

1. Jumping jacks
2. Wall sit/single leg dead lift
3. Push-up
4. Plank
5. Mountain climber
6. Squat
7. Triceps dip
8. Superman

Exercise 1 to 8 were performed sequentially with a 15-s rest between exercises. Completing all exercises (1 to 8) was considered one circuit, and two circuits were completed in each session, with a 3-min rest between circuits. To increase the load, the duration of each exercise was increased as follows^39,40^:

- Weeks 1 and 2: 10 s per exercise
- Weeks 3 and 4: 12 s per exercise
- Weeks 5 and 6: 14 s per exercise
- Weeks 7 and 8: 16 s per exercise

Before starting the exercise program, a 10-min warm-up, including light running and stretching exercises, was conducted, followed by a similar cool-down activity. The intensity of the exercise throughout the session was set at 65% of heart rate reserve (HRR), monitored using a Polar heart rate monitor (polar H10, USA). HRR was calculated by subtracting resting heart rate (RHR) from maximum heart rate (MHR). MHR considered as 207–0.7*age and RHR as the number of heart beats per minute while at complete rest, typically measured first thing in the morning^41^.

### Supplementation

Active treatment involved the oral administration of 8 mg of AST (Nature Vision; USA), sourced from H. pluvialis (microalgae). The inactive ingredients used for both the supplement and placebo included dicalcium phosphate, microcrystalline cellulose, stearic acid, silicon dioxide, and magnesium stearate. Participants in P, S, CT + P and CT + S were instructed to take one 8-mg tablet of either the placebo (which contained all the ingredients of the supplement except for AST) or the AST supplement immediately after lunch to maximize absorption, for a duration of 8 weeks.

### Dietary assessment

3-day food records were used before, during, and after the study period. It included details on the volume or weight and type of food consumed. Nutritionist IV software (Version 4.1, First Databank Division, The Hearst Corporation, and San Bruno, CA, USA) was used to evaluate energy and macronutrient intake. At each bi-weekly visit, participants’ adherence to the study protocol was monitored by counting the remaining AST tablets and reminding them to maintain their usual diet, lifestyle, and medication regimen.

### RNA extraction and quantitative real-time PCR

Total RNA was extracted using the GeneAll Kit (General Biosystems, Seoul, Korea, Cat# 315–150) in accordance with the manufacturer’s guidelines. Thermo Fisher Scientific Nanodrop was used to assess the RNA quality, measuring absorbance at wavelengths of 260 and 280 nm to determine RNA concentration in each sample. The A260/A280 ratio provides a general indication of nucleic acid (RNA) purity. Since the absorbance ratios for the samples were between 1.8 and 2.2, the RNA purity was confirmed. cDNA synthesis was then performed using Stem-loop quantitative reverse transcription PCR (RT-qPCR) (Bonyakhteh, Tehran, Iran, Cat# BON209001). Following this, the BONmiR QPCR Kit (Bonyakhteh, Tehran, Iran, Cat# BON209002) was employed to analyze miRNA gene expression using the Rotor Gene 6000 machine (Corbett, Concorde, NSW, Australia). The relative levels of miRNAs (fold changes) were calculated using the 2-ΔΔCt method (Livak). The changes in threshold cycle (ΔCt) were determined by subtracting the Ct values of the reference SNORD from the Ct values of the target miRNAs (miRNA-122, miRNA-126-5p, and miRNA-146a). ΔΔCt was then calculated by subtracting the average ΔCt values of the control group (healthy individuals) from those of the case group (pre-diabetes or diabetes). The formula used was ΔΔCt = (Ct miRNA − Ct SNORD) T2DM or pre-diabetes − (Ct miRNA − Ct SNORD) healthy individuals.

To assess the expression levels of miRNA-122, miRNA-126-5p, and miRNA-146a, qPCR was performed on whole blood samples, with SNORD serving as the internal control gene. Amplification efficiency, a critical factor for the accuracy and reliability of real-time qPCR, was calculated from the slope of the log-linear region, similar to that used for standard curves. The primer sets used in this study exhibited efficiencies between 90 and 110%.

### Bioinformatics analysis

Bioinformatics tools, including miRDB (http://www.mirdb.org), miRanda (http://www.microrna.org/microrna/), and TargetScan (http://www.targetscan.org), were utilized to identify the potential mRNA targets of miRNA-122, miRNA-126-3p, and miRNA-146a (Table 1).

### Metabolic indices measurement

The levels of fasting blood glucose (FBG), insulin, total cholesterol, low-density lipoprotein cholesterol (LDL-C), and high-density lipoprotein cholesterol (HDL-C) were measured using the Selectra Autoanalyzer (Pars Azmoon, Iran) with standard commercial kits. The following clinical definitions and reference ranges were considered: FBG measuring between 100 and 125 mg/dL indicates prediabetes, while 126 mg/dL or higher suggests diabetes mellitus. Regarding HbA1c, it indicates the plasma glucose level for the preceding 2–3 months, with pre-diabetes set for 5.7–6.4%. An increase of 6.5% and above indicates diabetes. Insulin Resistance: The Homeostasis Model Assessment of Insulin Resistance (HOMA-IR) was calculated using the formula:

$${\text{HOMA - IR = }}\frac{{{\text{Fasting}}\,{\text{Insulin}}\left( {\mu U/mL} \right) \times {\text{Fasting}}\,{\text{Gulcose}}\left( {mmol/L} \right)}}{22.5}$$

Insulin resistance is suggested with A HOMA-IR > 2.5. Lipid profile, cholesterol: average is < 200 mg/dL, LDL-C: optimal < 100 mg/dL, HDL-C: protective > 50 mg/dL for women, normal < 150 mg/dL for TG^42,43^.

These metabolic indicators were selected due to their relevance in evaluating the cardiometabolic and glycemic status of individuals with T2DM. HbA1c was measured using an Adichem device through the Ion Exchange Chromatography method and reported as a percentage.

### Inflammation measurement

To assess inflammation, we used ELISA to measure the serum levels of IL-36α, IL-36γ, IL-36Ra, and IL-17. The kits for these measurements were sourced from Cusabio Biotech Co., Ltd., and we conducted the experiments following the manufacturer’s guidelines. Each sample was tested twice and in a random order to ensure accuracy.

### Statistical analysis

We used descriptive statistics to summarize the participants’ characteristics and measurements. We checked the normality of the data using the Shapiro–Wilk test. To analyze differences within groups, we employed ANCOVA (using the pre-test as a covariate) followed by Bonferroni’s correction for post-hoc testing. All statistical analyses were conducted using SPSS V22 (SPSS Inc., Chicago, IL), with a significance level set at *p* < 0.05.

## Results

### Clinical and anthropometric characteristics of the study population

Table 2 details the participant’s baseline physical and medical characteristics. As indicated, no statistically significant difference was observed in basic characteristics. In addition, none of the patients took any special medicine for weight loss during the study.

### Expression levels of miRNA-122, miRNA-126-3p, and miRNA-146a

**Fig. 1:**
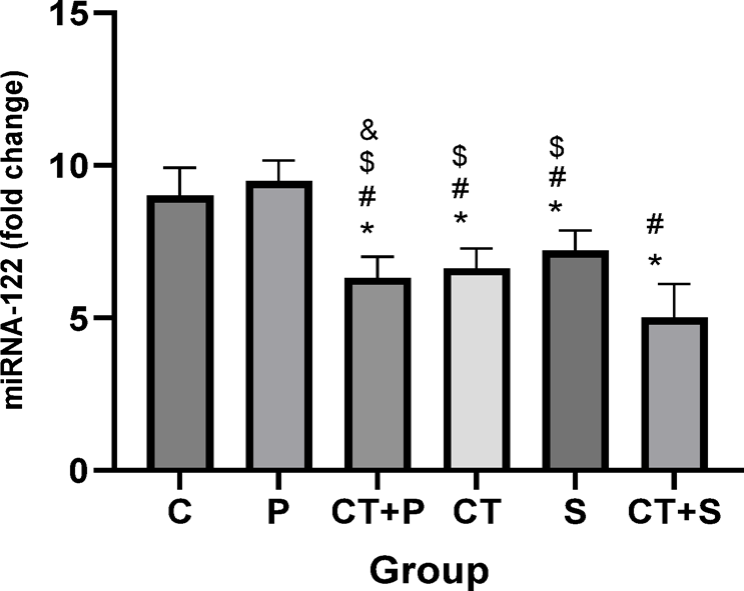
Comparison of the expression levels (Mean ± SD) of miRNA-122 in post-tests. # indicates a significant difference with the P. $ indicates a significant difference with the CT + S. & indicates a significant difference with the S. *C* control group, *P* placebo, *CT* + *P* combined training + placebo, *CT* combined training, *S* supplement, and *CT* + *S* combined training + supplement.

The actual values (Mean ± SD) for all measured variables are presented in supplementary table S1. The results indicated a significant difference between groups in miRNA-122 (F_(5,83)_ = 67.58, *p* < 0.001, η^2^ = 0.756), miRNA-126-3p (F_(5,83)_ = 21.69, *p* < 0.001, η^2^ = 0.623), and miRNA-146a (F_(5,83)_ = 27.35, *p* < 0.001, η^2^ = 0.677) expression levels, while adjusting for pre-test. The *post-hoc* test for miRNA-122 showed significantly lower levels in CT + P (d: 4.17, *p* < 0.001), CT (d: 3.52, *p* < 0.001), S (d: 2.74, *p* < 0.001), and CT + S (d: 5.77, *p* < 0.001) compared with CG. *Post-hoc* tests revealed significantly lower levels in CT + P (d: 4.44, *p* < 0.001), CT (d: 3.79, *p* < 0.001), S (d: 3.01, *p* < 0.001), and CT + S (d: 6.04, *p* < 0.001) compared with P. Also, significantly higher levels were observed in S (d: − 2.73, *p* = 0.013), and significantly lower in CT + S (d: 0.27, *p* = 0.004) compared with CT + P. Additionally, the CT + S group showed significantly lower levels (d: 2.25, *p* < 0.001) compared with CT group. A significant lower was observed in CT + S (d: 3.03, *p* < 0.001) compared with S group. No difference was found between the other groups (*p* > 0.05) (Fig. 1).

**Fig. 2:**
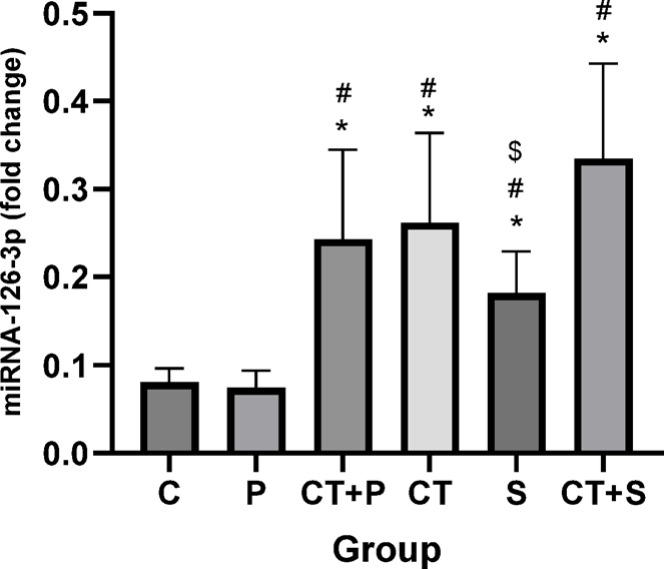
Comparison of the expression levels (Mean ± SD) of miRNA-126-3p in post-tests. # indicates a significant difference with the P. $ indicates a significant difference with the CT + S. *C* control group, *P* placebo, *CT* + *P* combined training + placebo, *CT* combined training, *S* supplement, and *CT* + *S* combined training + supplement.

The post hoc correction test for miRNA-126 revealed significantly higher levels in CT + P (d: − 2.11, *p* < 0.001), CT (d: − 2.36, *p* < 0.001), S (d: − 1.31, *p* = 0.030), and CT + S (d: − 3.29, *p* < 0.001) compared with C group. *Post-hoc* test revealed significantly higher levels in CT + P (d: − 2.21, *p* < 0.001), CT (d: − 2.46, *p* < 0.001), S (d: − 1.42, *p* = 0.015), and CT + S (d: − 3.39, *p* < 0.001) compared with P group. Additionally, miRNA-126 levels were significantly higher in CT + S (d: − 1.97, *p* < 0.001) compared with S group. No significant differences were observed between the other groups (*p* > 0.05) (Fig. 2).

**Fig. 3:**
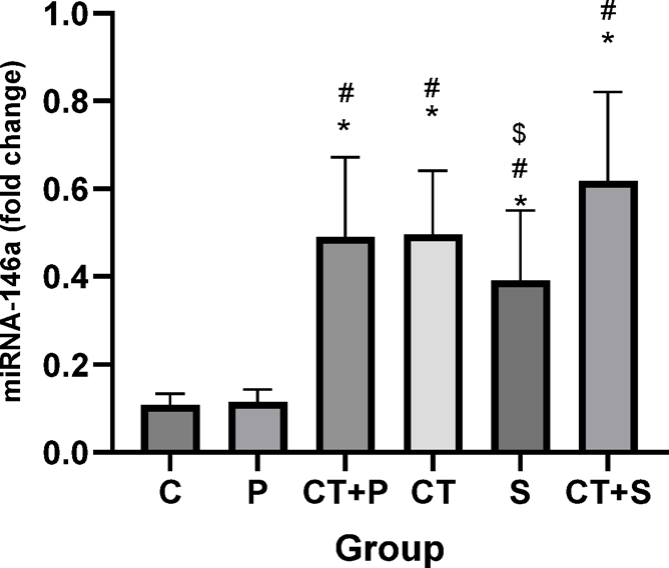
Comparison of the expression levels (Mean ± SD) of miRNA-146a in post-tests. # indicates a significant difference with the P. $ indicates a significant difference with the CT + S. *C* control group, *P* placebo, *CT* + *P* combined training + placebo, *CT* combined training, *S* supplement, and *CT* + *S* combined training + supplement.

Bonferroni’s *post-hoc* correction test for miRNA-146a revealed significantly higher levels in CT + P (d: − 2.68, *p* < 0.001), CT (d: − 2.72, *p* < 0.001), S (d: − 2.04, *p* < 0.001), and CT + S (d: − 3.67, *p* < 0.001) compared with C group. *Post-hoc* test showed significantly higher levels in CT + P (d: − 2.68, *p* < 0.001), CT (d: − 2.72, *p* < 0.001), S (d: − 2.04, *p* < 0.001), and CT + S (d: − 3.67, *p* < 0.001) compared with P group. Additionally, miRNA-146a levels were significantly lower in the CT + S (d: − 1.63, *p* = 0.003) compared with S group. No significant differences were observed between the other groups (*p* > 0.05) (Fig. 3).

### IL-36α, IL-36γ, IL-36Ra, and IL-17 levels

**Fig. 4:**
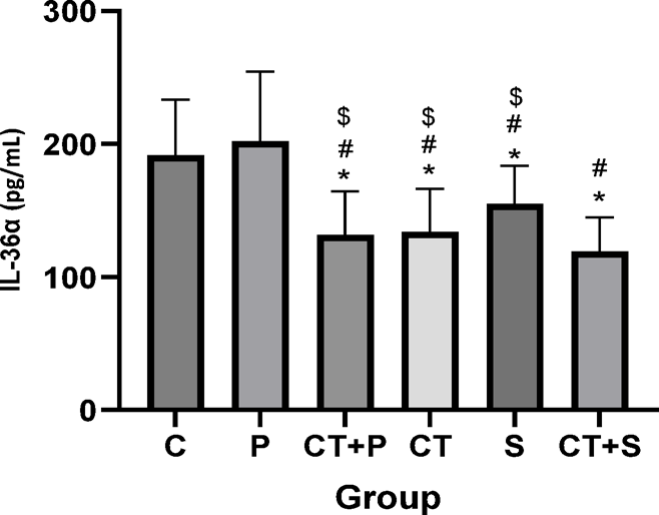
Serum IL-36α level (Mean ± SD) in post-test. * indicates a significant difference with the C. # indicates a significant difference with the P. $ indicates a significant difference with the CT + S. *C* control group, *P* placebo, *CT* + *P* combined training + placebo, *CT* combined training, *S* supplement, and *CT* + *S* combined training + supplement.

The results of the univariate ANCOVA test showed a significant difference between groups in IL-36α (F_(5,83)_ = 24.28, *p* < 0.001, η^2^ = 0.453), IL-36γ (F_(5,83)_ = 24.38, *p* < 0.001, η^2^ = 0.537), IL-36Ra (F_(5,83)_ = 27.88, *p* < 0.001, η^2^ = 0.471), and IL-17 (F_(5,83)_ = 17.87, *p* < 0.001,η^2^ = 0.434) levels, while adjusting for pre-training. Bonferroni’s *post-hoc* correction test for IL-36α revealed significantly lower levels in CT + P (d: 1.95, *p* < 0.001), CT (d: 2.42, *p* < 0.001), S (d: 1.95, *p* < 0.001), and CT + S (d: 3.29, *p* < 0.001) compared with C group. *Post-hoc* test revealed significantly lower levels in CT + P (d: 2.30, *p* < 0.001), CT (d: 2.77, *p* < 0.001), S (d: 2.31, *p* < 0.001), and CT + S (d: 3.64, *p* < 0.001) compared with P. Additionally, IL-36α were significantly lower levels in CT + S compared with CT + P (d:1.34, *p* = 0.031), CT (d:1.24, *p* = 0.043), and S (d:1.33, *p* = 0.025). No significant differences were observed between the other groups (*p* > 0.05) (Fig. 4).

**Fig. 5:**
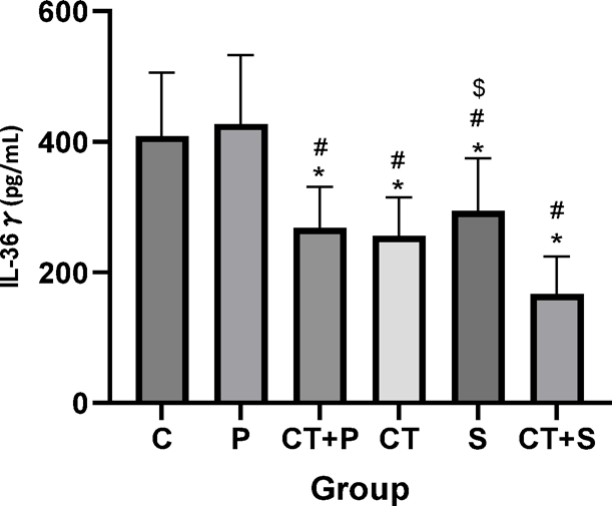
Serum IL-36γ level (Mean ± SD) in post-test. * indicates a significant difference with the C. # indicates a significant difference with the P. $ indicates a significant difference with the CT + S. *C* control group, *P* placebo, *CT* + *P* combined training + placebo, *CT* combined training, *S* supplement, and *CT* + *S* combined training + supplement.

Bonferroni’s *post-hoc* correction test for IL-36γ revealed significantly lower levels in CT + P (d: 2.43, *p* < 0.001), CT (d: 2.42, *p* < 0.001), S (d: 1.44, *p* = 0.012), and CT + S (d: 3.36, *p* < 0.001) compared with C group. The *Post-hoc* test revealed significantly lower levels in CT + P (d: 2.59, *p* < 0.001), CT (d: 2.58, *p* < 0.001), S (d: 1.60, *p* = 0.004), and CT + S (d: 3.52, *p* < 0.001) compared with P group. Also, the *post-hoc* test showed significantly lower levels in CT + S (d: 1.33, *p* = 0.025) compared with S. No difference was found between the other groups (*p* > 0.05) (Fig. 5).

**Fig. 6:**
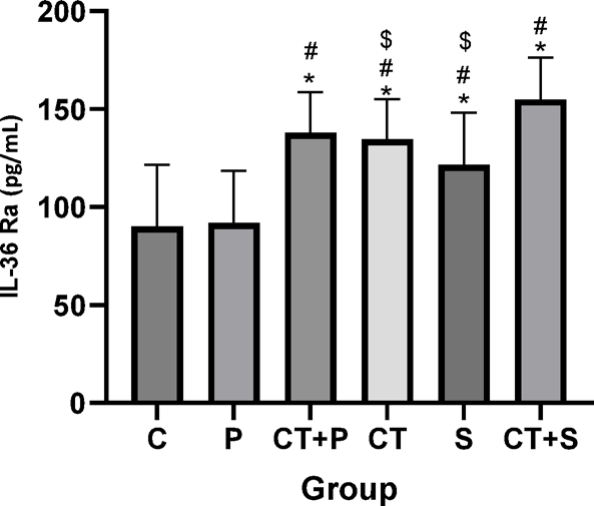
Serum IL-36Ra level (Mean ± SD) in post-test. * indicates a significant difference with the C. # indicates a significant difference with the P. $ indicates a significant difference with the CT + S. *C* control group, *P* placebo, *CT* + *P* combined training + placebo, *CT* combined training, *S* supplement, and *CT* + *S* combined training + supplement.

Bonferroni’s *post-hoc* correction test for IL-36Ra revealed significantly higher levels in CT + P (d: − 2.52, *p* < 0.001), CT (d: − 2.46, *p* < 0.001), S (d: − 2.25, *p* < 0.001), and CT + S (d: − 3.68, *p* < 0.001) compared with C group. The *Post-hoc* test showed significantly higher levels in CT + P (d: − 2.72, *p* < 0.001), CT (d: − 2.35, *p* < 0.001), S (d: − 2.44, *p* < 0.001), and CT + S (d: − 3.88, *p* < 0.001) compared with P group. Also, IL-36Ra were significantly higher levels in CT + S (d: − 2.83, *p* = 0.006) compared with CT group. The *post-hoc* test revealed significantly higher levels difference in CT + S (d: − 1.43, *p* = 0.014) compared with S. No difference was found between the other groups (*p* > 0.05) (Fig. 6).

**Fig. 7:**
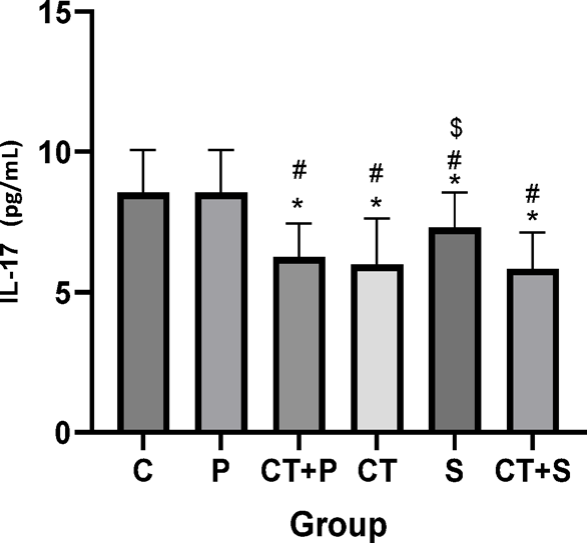
Serum IL-17 level (Mean ± SD) in post-test. * indicates a significant difference with the C. # indicates a significant difference with the P. $ indicates a significant difference with the CT + S. *C* control group, *P* placebo, *CT* + *P* combined training + placebo, *CT* combined training, *S* supplement, and *CT* + *S* combined training + supplement.

Bonferroni’s *post-hoc* correction test for IL-17 revealed significantly lower levels in CT + P (d: 2.18, *p* < 0.001), CT (d: 2.46, *p* < 0.001), S (d: 1.39, *p* = 0.017), and CT + S (d: 2.90, *p* < 0.001) compared with C group. *Post-hoc* test showed significantly lower in CT + P (d: 2.03, *p* < 0.001), CT (d: 2.32, *p* < 0.001), S (d: 1.24, *p* = 0.050), and CT + S (d: 2.75, *p* < 0.001) compared with P group. Also, Bonferroni’s correction *post-hoc* test revealed significantly lower levels in CT + S (d: 1.51, *p* = 0.007) compared to S group. No difference was found between the other groups (*p* > 0.05) (Fig. 7).

### Total antioxidant capacity (TAC), superoxide dismutase (SOD) and glutathione peroxidase (GPx) activity

**Fig. 8:**
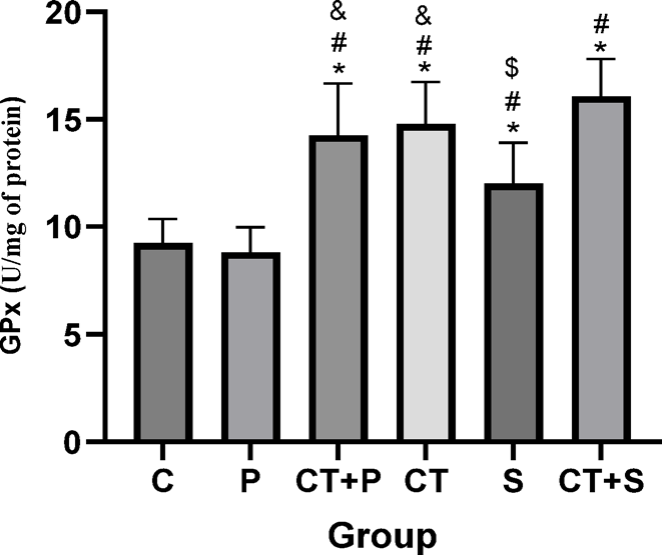
GPx level (Mean ± SD) in post-test. * indicates a significant difference with the C. # indicates a significant difference with the P. $ indicates a significant difference with the CT + S. & indicates a significant difference with the S. *C* control group, *P* placebo, *CT* + *P* combined training + placebo, *CT* combined training, *S* supplement, and *CT* + *S* combined training + supplement.

The univariate ANCOVA test showed a significant difference between groups in TAC (F_(5,83)_ = 44.51, *p* < 0.001, η^2^ = 0.729), SOD (F_(5,83)_ = 9.87, *p* < 0.001, η^2^ = 0.338), and GPx (F_(5,83)_ = 38.06, *p* < 0.001, η^2^ = 0.629) levels, while adjusting for the pre-training. Bonferroni’s *post-hoc* test for GPx revealed significantly higher levels in CT + P (d: − 3.11, *p* < 0.001), CT (d: − 3.27, *p* < 0.001), S (d:—1.73, *p* = 0.001), and CT + S (d: − 4.13, *p* < 0.001) compared with C group. Post hoc test showed significantly higher levels in CT + P (d: − 3.27, *p* < 0.001), CT (d: − 3.48, *p* < 0.001), S (d: − 1.94, *p* < 0.001), and CT + S (d: − 4.34, *p* < 0.001) compared with P group. Also, GPx were significantly lower levels in S (d: 1.37, *p* = 0.019) compared to CT + P and CT (d: 1.54, *p* = 0.006) groups. The Post hoc test showed significantly higher levels in CT + S compared with S (d: − 2.40, *p* < 0.001). No difference was found between the other groups (*p* > 0.05) (Fig. 8).

**Fig. 9:**
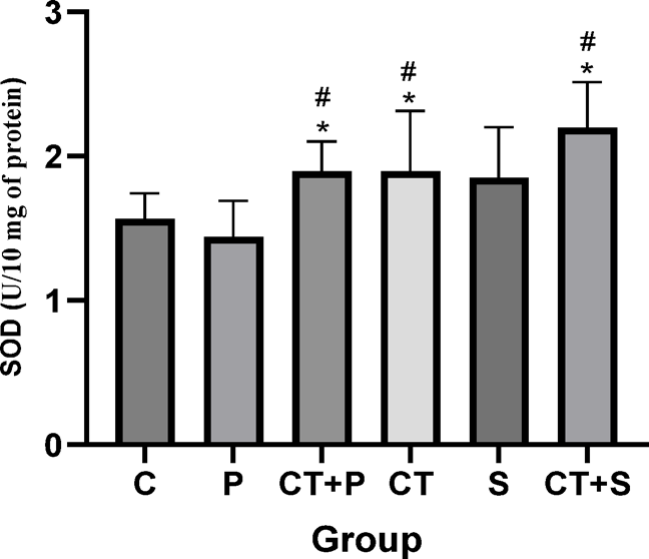
SOD level (Mean ± SD) in post-test. * indicates a significant difference with the C. # indicates a significant difference with the P. *C* control group, *P* placebo, *CT* + *P* combined training + placebo, *CT* combined training, *S* supplement, and *CT* + *S* combined training + supplement.

Bonferroni’s *post-hoc* test for SOD revealed significantly higher levels in CT + P (d: − 1.30, *p* = 0.033), CT (d: − 1.54, *p* = 0.006), and CT + S (d: − 2.23, *p* < 0.001) compared with C group. The *Post-hoc* test showed significantly higher levels in CT + P (d: − 1.43, *p* = 0.013), CT (d: − 1.68, *p* = 0.002), and CT + S (d: − 2.36, *p* < 0.001) compared with P group. No difference was found between the other groups (*p* > 0.05) (Fig. 9).

**Fig. 10:**
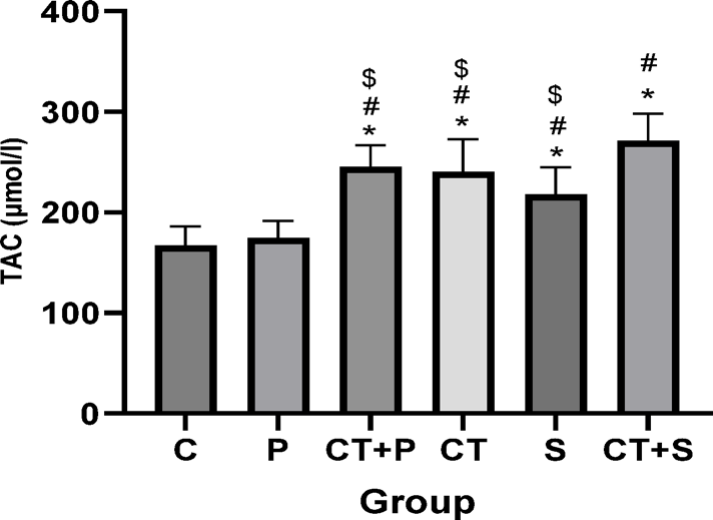
TAC level (Mean ± SD) in post-test. * indicates a significant difference with the C. # indicates a significant difference with the P. $ indicates a significant difference with the CT + S. *C* control group, *P* placebo, *CT* + *P* combined training + placebo, *CT* combined training, *S* supplement, and *CT* + *S* combined training + supplement.

Bonferroni’s *post-hoc* correction test for TAC revealed significantly higher levels in CT + P (d: − 3.34, *p* < 0.001), CT (d: − 3.25, *p* < 0.001), S (d: − 2.20, *p* = 0.001), and CT + S (d: − 4.78, *p* < 0.001) compared with C group. The *Post-hoc* test revealed significantly higher levels in CT + P (d: − 3.30, *p* < 0.001), CT (d: − 3.20, *p* < 0.001), S (d: − 2.16, *p* < 0.001), and CT + S (d: − 4.74, *p* < 0.001) compared with P group. Also, TAC were There were significantly higher in CT + S compared with S (d: − 2.57, *p* < 0.001), CT (d: − 1.53, *p* = 0.006), CT + P (d: − 1.43, *p* = 0.013) groups. No difference was found between the other groups (*p* > 0.05) (Fig. 10).

### Serum lipid profiles, fasting blood glucose, insulin resistance, HbA1c, and humanin levels

The univariate ANCOVA test showed significant differences between groups in cholesterol (F_(5,83)_ = 12.21, *p* < 0.001, η^2^ = 0.287), triglycerides (TG) (F_(5,83)_ = 23.29, *p* < 0.001, η^2^ = 0.591), HDL (F_(5,83)_ = 22.32, *p* < 0.001, η^2^ = 0.382), fasting insulin (F_(5,83)_ = 44.51, *p* < 0.001, η^2^ = 0.729), HbA1c (F_(5,83)_ = 30.21, *p* < 0.001, η^2^ = 0.677), fasting blood glucose (F_(5,83)_ = 45.39, *p* < 0.001, η^2^ = 0.777), insulin resistance (F_(5,83)_ = 15.32, *p* < 0.001, η^2^ = 0.281), and humanin (F_(5,83)_ = 24.29, *p* < 0.001, η^2^ = 0.460) levels, while adjusting for the pre-training.

**Fig. 11:**
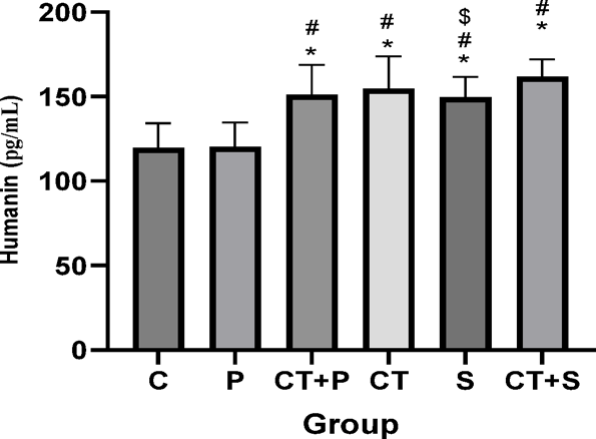
Serum humanin level ((Mean ± SD). * Indicates a significant difference with the C. # indicates a significant difference with the P. $ indicates a significant difference with the CT + S. *C* control group, *P* placebo, *CT* + *P* combined training + placebo, *CT* combined training, *S* supplement, and *CT* + *S* combined training + supplement.

Bonferroni’s *post-hoc* correction test for humanin revealed significantly higher in CT + P (d: − 2.69, *p* < 0.001), CT (d: − 2.77, *p* < 0.001), S (d: − 1.86, *p* = 0.001), and CT + S (d: − 3.18, *p* < 0.001) compared with C group. The *Post-hoc* test showed significantly higher in CT + P (d: − 2.74, *p* < 0.001), CT (d:—2.88, *p* < 0.001), S (d: − 1.91, *p* < 0.001), and CT + S (d: − 3.23, *p* < 0.001) compared with P group. Also, humanin were significantly higher in CT + S compared with S group (d: − 1.31, *p* = 0.030). No difference was found between the other groups (*p* > 0.05) (Fig. 11).

**Fig. 12:**
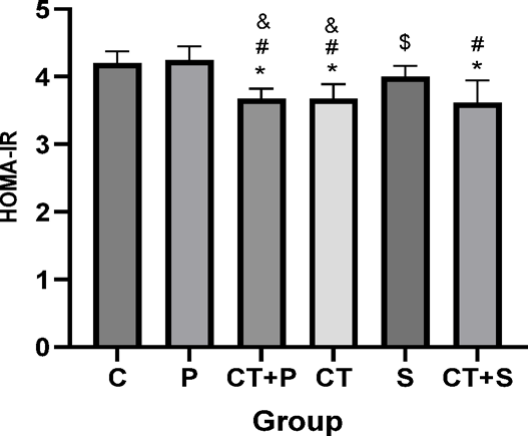
Serum insulin resistance level (Mean ± SD). * Indicates a significant difference with the C. # indicates a significant difference with the P. $ indicates a significant difference with the CT + S. & indicates a significant difference with the S. *C* control group, *P* placebo, *CT* + *P* combined training + placebo, *CT* combined training, *S* supplement, and *CT* + *S* combined training + supplement.

Bonferroni’s *post-hoc* test for HOMA-IR showed significantly lower in CT + P (d: 3.29, *p* < 0.001), CT (d: 3.32, *p* < 0.001), and CT + S (d: 3.46, *p* < 0.001) compared to C group. *Post-hoc* test revealed significantly lower in CT + P (d: 3.52, *p* < 0.001), CT (d: 3.55, *p* < 0.001), and CT + S (d: 3.70, *p* < 0.001) compared with P group. The *post-hoc* test showed significantly lower in S compared to CT + P (d:—2.39, *p* < 0.001), and CT (d:—2.42, *p* < 0.001) groups. Also, HOMA-IR were significantly lower in CT + S compared with S (d: 2.56, *p* = 0.030) group. No difference was found between the other groups (*p* > 0.05) (Fig. 12).

**Fig. 13:**
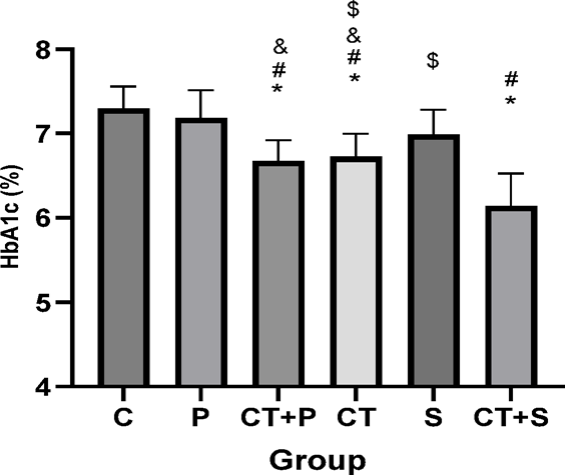
Serum HbA1c level (Mean ± SD). * Indicates a significant difference with the C. # indicates a significant difference with the P. $ indicates a significant difference with the CT + S. & indicates a significant difference with the S. *C* control group, *P* placebo, *CT* + *P* combined training + placebo, *CT* combined training, *S* supplement, and *CT* + *S* combined training + supplement.

Bonferroni’s *post-hoc* test for HbA1c showed significantly lower in CT + P (d: 2.43, *p* < 0.001), CT (d: 2.20, *p* < 0.001), and CT + S (d: 4.24, *p* < 0.001) compared to C group. The *Post-hoc* test showed significantly lower in CT + P (d: 2.12, *p* < 0.001), CT (d: 1.89, *p* < 0.001), and CT + S (d: 3.93, *p* < 0.001) compared with P group. The *post-hoc* test showed significantly higher in S compared with CT + P (d: − 1.43, *p* = 0.014), and CT (d: − 1.19, *p* = 0.049) groups. Also, HbA1c were significantly lower in CT + S compared with S (d: 3.24, *p* < 0.001), and CT (d: 2.04, *p* < 0.001) groups. No difference was found between the other groups (*p* > 0.05) (Fig. 13).

**Fig. 14:**
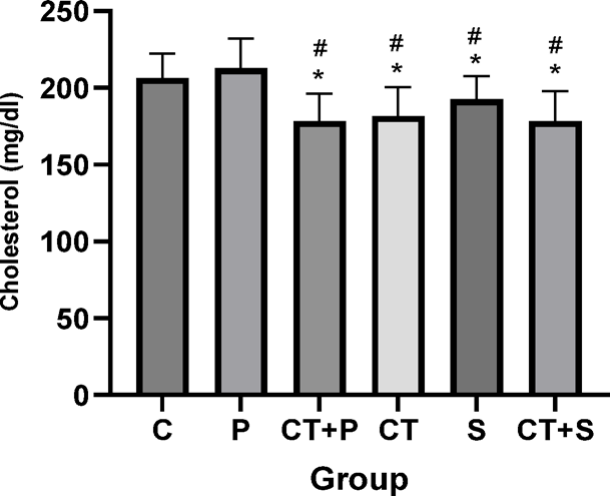
Serum cholesterol level levels (Mean ± SD). * Indicates a significant difference with the C. # indicates a significant difference with the P. *C* control group, *P* placebo, *CT* + *P* combined training + placebo, *CT* combined training, *S* supplement, and *CT* + *S*combined training + supplement.

Bonferroni’s *post-hoc* test for cholesterol showed significantly lower in CT + P (d: 2.05, *p* < 0.001), CT (d: 1.96, *p* < 0.001), S (d: 1.36, *p* = 0.022), and CT + S (d: 2.02, *p* < 0.001) compared with C group. *Post-hoc* test showed significantly lower in CT + P (d: 2.16, *p* < 0.001), CT (d: 2.03, *p* < 0.001), S (d: 1.46, *p* = 0.009), and CT + S (d: 2.13, *p* < 0.001) compared with P group. No difference was found between the other groups (*p* > 0.05) (Fig. 14).

**Fig. 15:**
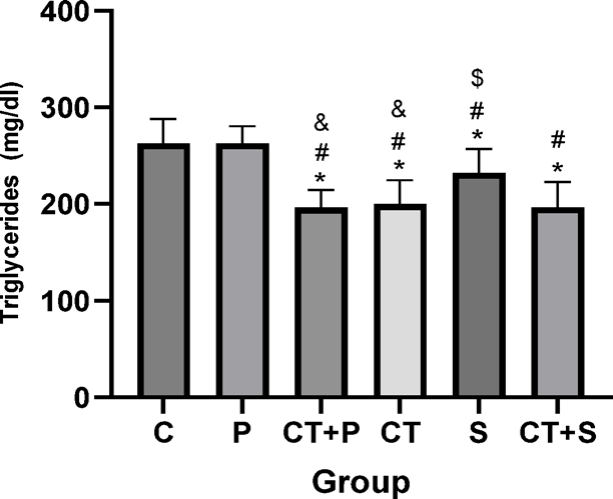
Serum triglycerides level (Mean ± SD). * Indicates a significant difference with the C. # indicates a significant difference with the P. $ indicates a significant difference with the CT + S. & indicates a significant difference with the S. *C* control group, *P* placebo, *CT* + *P* combined training + placebo, *CT* combined training, *S* supplement, and *CT* + *S* combined training + supplement.

Bonferroni’s correction *post-hoc* test for TG showed significantly lower in CT + P (d: 2.81, *p* < 0.001), CT (d: 2.80, *p* < 0.001), S (d: 1.41, *p* = 0.014), and CT + S (d: 3.01, *p* < 0.001) compared with C group. *Post-hoc* test showed significantly lower in CT + P (d: 2.76, *p* < 0.001), CT (d: 2.75, *p* < 0.001), S (d: 1.36, *p* = 0.021), and CT + S (d: 2.95, *p* < 0.001) compared with P group. The TG were significantly higher in S compared to CT + P (d: − 1.40, *p* = 0.020) and CT (d: − 1.38, *p* = 0.018) groups. Also, the *Post-hoc* test showed significantly lower in CT + S compared with S (d: 1.59, *p* = 0.003). No difference was found between the other groups (*p* > 0.05) (Fig. 15).

**Fig. 16:**
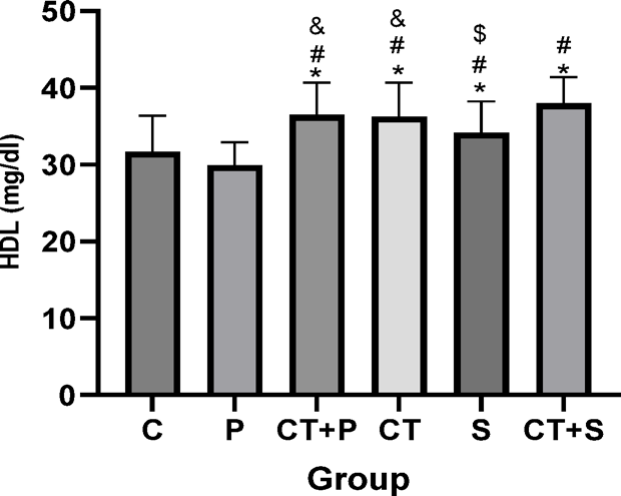
Serum HDL level (Mean ± SD). * Indicates a significant difference with the C. # indicates a significant difference with the P. $ indicates a significant difference with the CT + S. & indicates a significant difference with the S. *C* control group, *P* placebo, *CT* + *P* combined training + placebo, *CT* combined training, *S* supplement, and *CT* + *S* combined training + supplement.

Bonferroni’s correction *post-hoc* test for HDL showed significantly higher in CT + P (d: − 2.64, *p* < 0.001), CT (d: − 2.74, *p* < 0.001), S (d: − 1.31, *p* = 0.032), and CT + S (d: − 2.83, *p* < 0.001) compared with C group. The *Post-hoc* test showed significantly higher in CT + P (d: − 2.71, *p* < 0.001), CT (d: − 2.80, *p* < 0.001), S (d: − 1.37, *p* = 0.021), and CT + S (d: − 2.89, *p* < 0.001) compared with P group. The *Post-hoc* test showed significantly lower in S compared to CT + P (d: 1.33, *p* = 0.026) and CT (d: 1.43, *p* = 0.013). Also, HDL were significantly higher in CT + S compared with S group (d: − 1.52, *p* = 0.006). No difference was found between the other groups (*p* > 0.05) (Fig. 16).

**Fig. 17:**
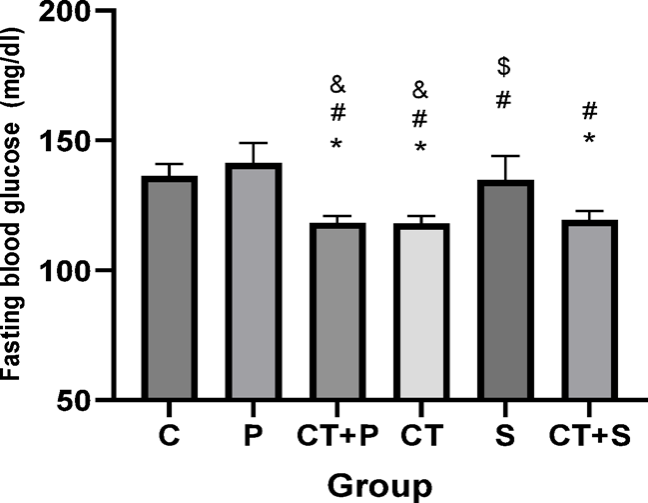
Serum Fasting Blood Glucose level (Mean ± SD). * Indicates a significant difference with the C. # indicates a significant difference with the P. $ indicates a significant difference with the CT + S. & indicates a significant difference with the S. *C* control group, *P* placebo, *CT* + *P* combined training + placebo, *CT* combined training, *S* supplement, and *CT* + *S* combined training + supplement.

Bonferroni’s *post-hoc* correction test for Fasting Blood Glucose showed significantly lower in CT + P (d: 3.44, *p* < 0.001), CT (d: 3.50, *p* < 0.001), and CT + S (d: 3.29, *p* < 0.001) compared with C group. The *Post-hoc* test showed significantly lower in CT + P (d: 4.21, *p* < 0.001), CT (d: 4.27, *p* < 0.001), S (d: 1.25, *p* = 0.047), and CT + S (d: 4.05, *p* < 0.001) compared with P group. Also, the *Post-hoc* test showed significantly higher in S compared with CT + P (d: − 2.95, *p* = 0.030) and CT (d: − 3.01, *p* = 0.030). Also, the FBG were significantly lower in CT + S compared with S group (d: 2.81, *p* < 0.001). No difference was found between the other groups (*p* > 0.05) (Fig. 17).

**Fig. 18:**
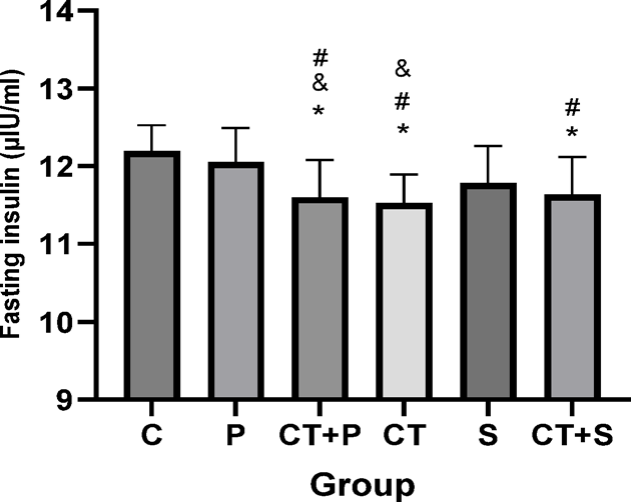
Serum fasting insulin level (Mean ± SD). * Indicates a significant difference with the C. # indicates a significant difference with the P. $ indicates a significant difference with the CT + S. & indicates a significant difference with the S. *C* control group, *P* placebo, *CT* + *P* combined training + placebo, *CT* combined training, *S* supplement, and *CT* + *S* combined training + supplement.

Bonferroni’s *post-hoc* correction test for Fasting insulin showed significantly lower in CT + P (d: 2.24, *p* < 0.001), CT (d: 2.73, *p* < 0.001), and CT + S (d: 2.20, *p* < 0.001) compared with C group. The *Post-hoc* test showed significantly lower in CT + P (d: 1.95, *p* < 0.001), CT (d: 1.06, *p* = 0.045), and CT + S (d: 1.56, *p* = 0.005) compared with P group. Also, Fasting insulin showed significantly higher in S compared with CT + P (d: − 2.95, *p* = 0.030) and CT (d: − 3.01, *p* = 0.030) groups. No difference was found between the other groups (*p* > 0.05) (Fig. 18).

## Discussion

**Fig. 19:**
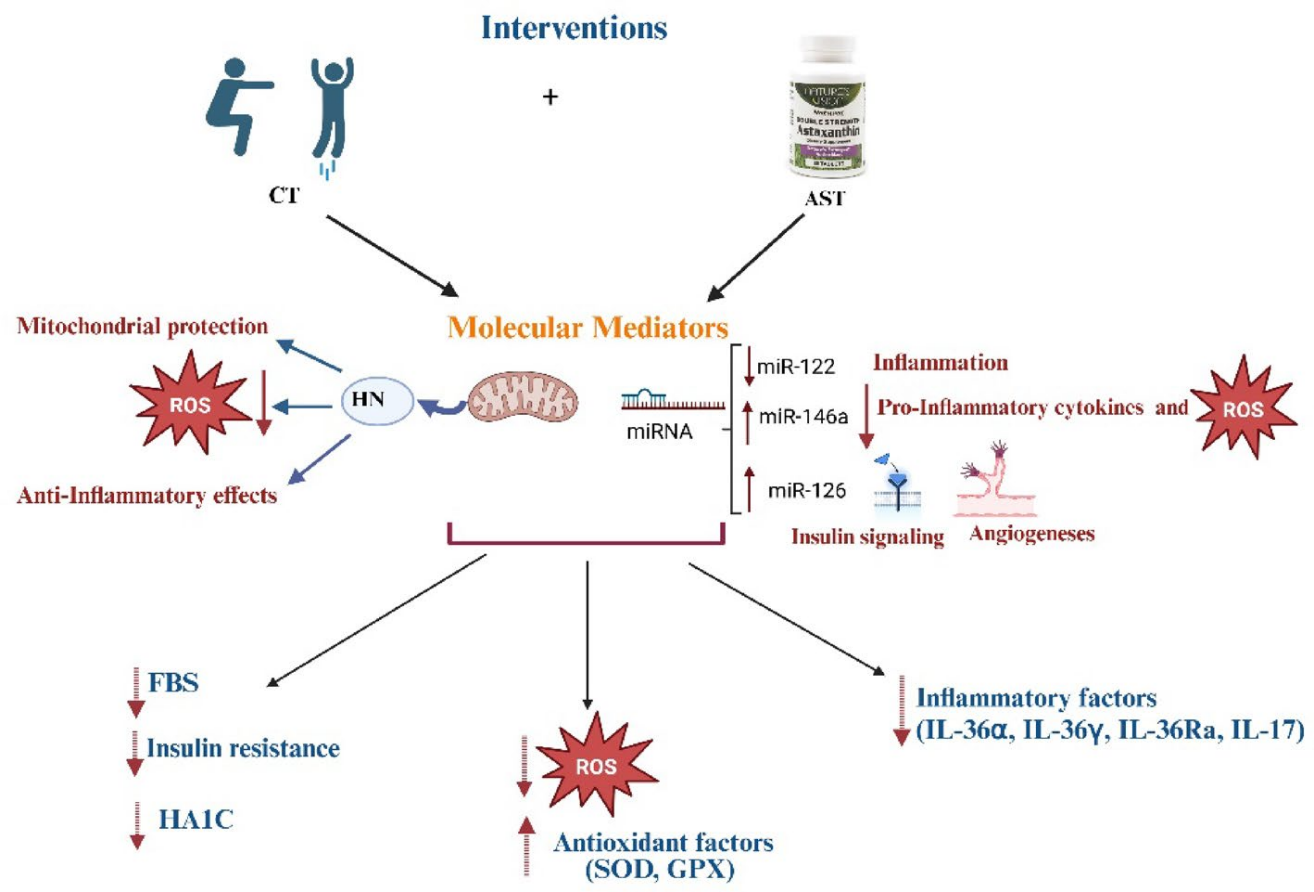
Effect of  - exercise and Astaxanthin supplementation on redox-sensetive miRNAs, Humanin, glucose metabolism oxidative stress and inflammation in type 2 diabetes .

The aim of this study was to investigate whether HN and miRNAs-122, -126-3p, and -146a could mediate the reduction of OS and IF induced by CT and AST supplementation in women with T2DM, potentially leading to improved diabetic symptoms and complications. Our results showed that CT and AST supplementation improved antioxidant defense and their combination were more effective than either intervention alone. CT and AST supplementation also reduced IF, with the combination again being more effective. CT and AST supplementation also increase blood concentration of HN, and their combination showed greater effects than AST supplementation, but not CT. In addition, CT and AST supplementation increased blood levels of miRNAs-126-3p, and -146a and decreased miRNA-122 with their combination being slightly more effective in decreasing miRNA-122. Both interventions also improved lipid profile, with their combination being more effective in improving HDL and TG levels, although not cholesterol. Furthermore, FBG, IR, and HbA1c were reduced by CT but not by AST supplementation (Fig. 19).

Elevated OS can lead to cellular damage and dysfunction, which exacerbates IR and contributes to the development of diabetic complications. OS could also activates inflammatory pathways, resulting in chronic IF that further disrupts normal metabolic processes and accelerates IR^5,44^. Our results showed that while TAC and serum levels of GPx were increased by both CT and AST supplementation, their combination led to more significant increases. Serum levels of SOD increased only with CT. Other studies also showed improved antioxidant defense in patients with T2DM following resistance training^45,46^, endurance training^47,48^high-intensity interval training (HIIT)^45^, TRX training^46^, and acute exercise^49^. Xu et al. reported that treating rats with type 1 diabetes with 10, 20, and 40 mg/kg AST for 5 days increased levels of GSH and SOD while reducing MDA, with the highest effect seen at 40 mg/kg^50^. Other studies also confirmed the antioxidant effects of AST in diabetic patients^21,51,52^. In addition, a systematic review and meta-analysis, published recently, concluded that AST supplementation could lead to increase TAC in athletes and improve cycling time trail performance^53^.

### Effect of CT and AST on OS, IF and HN

Reducing OS could also relieve IF, as confirmed by our results. Both interventions reduced the levels of pro-inflammatory markers (e.g., IL-36α and IL-36γ) and increased the levels of anti-inflammatory markers (e.g., IL-36 Ra). Given that both aerobic and resistance training individually lead to beneficial changes in inflammatory markers in individuals with T2DM and/or obesity, combining them seems to be the most effective exercise strategy for those with metabolic health issues^12^. The integration of aerobic and resistance exercise elicits potent anti-inflammatory effects by modulating adipose tissue metabolism and reducing pro-inflammatory cytokines^13^. A recently published systematic review and meta-analysis by Khalafi et al,. concluded that CT is superior to resistance training and HIIT in reducing systemic IF in T2D patients^54^. Papagianni et al. showed a significant improvement in IF after aerobic training^10^. Decreased serum levels of IL-17 after 8 weeks of aerobic training (Burce protocol) in men with T2DM has been reported by Alikhazaeil et al.^55^. However, another study^56^ did not find an anti-inflammatory effect after concurrent training in sedentary obese participants. Differences in participant characteristics (obese in their study vs. T2DM patients in ours) and variations in exercise protocols could possibly explain this controversy. Other studies^57,58^ aligned with our findings reported the anti-inflammatory effects of AST in diabetes. Chang et al.^20^ highlighted the pathways that might be involved in anti-inflammatory effect of AST including PI3K/AKT, Nrf2, NF-κB, ERK1/2, JNK, p38 MAPK, and JAK-2/STAT-3.

Moin et al. reported that HN exhibited antioxidant and anti-inflammatory effects in diabetic rats^59^. HN administration could improve several diabetes symptoms including HbA1c levels, FBG, IR, and lipid profiles. Physiologically, HN is produced in various organs such as the kidneys, skeletal muscles, brain, heart, and liver, and is then secreted into the bloodstream^22^. It protects cells against diseases strongly linked to OS and mitochondrial dysfunction^22^. Exercise is a source of physiological stressor of mitochondria stimulating adaptive response such as HN^60^. Resistance training (three times per week for twelve weeks with progressively increasing intensity) raised serum HN levels in men with impaired glucose metabolism, ultimately improving IR^61^. HIIT^11^ and endurance training^26^ have also been shown to increase HN serum levels. Studies showed that CT could be stronger stimulation than endurance and resistance training for HN secretion because it expose mitochondria to both endurance related and load related stress promoting maximal mitochondria adaptation and subsequently HN secretion^62^. Our results indicate that CT and ASX supplementation improved HN levels, with their combination leading to higher HN levels than AST alone, though not more than CT alone.

### Effect of CT and AST on MiRNAs-122, 126-3p and -146a

MiRNA-122, primarily expressed in the liver, suppressed TNF-α and IL-6 in human liver organoid models^63^. Studies^64,65^ have indicated that HIIT may serve as a non-pharmacological intervention to stimulate miRNA-122 secretion. However, Zolfi et al., showed that HIIT reduced MiRNA-122 in middle-aged men with metabolic syndrome^65^. These controversial results indicate that the effect of exercise on MiRNA-122 is complicated and context dependent. So, we chose CT trying to improve the consistency as it includes both kinds of exercise. We observed that miRNA-122 levels decreased in the CT, S, and CT + S groups, with the lowest levels in the CT + S group.

MiRNAs-126-3p and -146a, which are also involved in antioxidant defense, increased after CT and AST supplementation. The combination of these interventions led to higher levels of these miRNAs compared to AST alone, but not more than CT alone. MiRNA-126-3p is expressed exclusively in endothelial cells within capillaries and larger blood vessels^66^ and regulates IF by targeting various transcripts^67^. Overexpression of miRNA-126-3p has been shown to reduce IF in diabetic patients^30^. While previous studies^36,38^ have shown increased miRNA-126–3-p expression following aerobic training, resistance training alone is seems not to increase blood levels of miRNA-126-3p in diabetic patients^68^. The reason could be the main origin of miRNA-126–3-p which is endothelial cells. Because aerobic exercise increase blood flow and shear stress more than resistance exercise, it could provide stronger stimulation for miRNA-126–3-p secretion^68^. miRNA-146a, located on the long arm of chromosome 5^69^, has been found to play a protective role against IF by targeting genes involved in the inflammatory process^32^. Cirilli et al.^70^ found that high-intensity training downregulated miRNA-146a. However, other studies reported upregulation of miRNA-146a following resistance training^71^ and after 8 weeks of combined resistance training and HIIT^72^ in diabetic patients. These positive effects were observed with different exercise regimens (same day vs. different days)^72^. The variation in results may be attributed to differences in exercise protocols (aerobic training in Cirilli et al. vs. CT in Ghodrat et al. and our study).

The only study that explored the impact of AST on miRNAs in diabetes was conducted by Shokri-Mashhadi et al.^73^. They found that administering 8 mg/day of oral AST for 8 weeks reduced circulating levels of miRNA-146a but did not affect miRNA-126. It is possible that differences in participant sex (male and female participants in their study vs. only females in ours) may explain why they did not observe a significant effect of AST on miRNA-126, as men might require higher doses of AST to see significant changes.

### Effect of CT and AST on lipid profile, IR, HbA1c and FBG

Our data suggest that combining exercise with AST supplementation might improve oxidative status and IF through mechanisms involving HN and miRNAs 122, 126-3p, and 146a. Alleviating OS and IF could, in turn, lead to improvements in lipid profiles (e.g., TG, and HDL), IR, and reductions in HbA1c and FBG, as observed in our study. Furthermore, the combined approach seems to be more effective at improving cholesterol and TG levels.

A recent review by Al-Mhanna et al.^13^ concluded that CT likely enhances cardio metabolic health by improving insulin sensitivity, increasing muscle glucose uptake, and promoting favorable changes in body composition, including reduced adiposity. We also believe that mitigating OS and IF, potentially through HN and miRNAs, could contribute to these benefits. Different mechanisms can facilitate aerobic exercise effect of cardiometabolic health such as increase energy expenditure and promoting fat loos, enhancing lipoprotein lipase (LPL) activity, and improving IR. But other mechanisms mediate resistance effect of cardiometabolic health including increase basal metabolic rate due to increase muscle mass, direct reduction of cholesterol and LDL, enhances muscle glucose uptake and increase LPL activity^74^. We believe that CT could induce all of the aerobic and resistance effects.

CT has been shown to improve various glucose metabolism parameters, including HOMA-IR and FBG, in healthy adults^14^. Previous studies^12,75^ are consistent with our findings, indicating that CT improves IR and reduces FBG. Additionally, the reduction in HbA1c resulting from CT has been confirmed by multiple studies^8,76^.

Previous researches have also shown reductions in total cholesterol^72^, LDL^72^ and increases in HDL. Tai chi has also been shown to improve lipid profiles in patients with T2D^77^. Bassi et al.^78^ reported a decrease in LDL/HDL ratios after exercise training. Variations in exercise protocols, participant characteristics, and disease status might explain why some studies did not find significant changes in certain variables.

Additionally, the positive effects of AST on HbA1c^79^, HOMA-IR^17,51,79^, FBG^17,51^, and lipid profiles^17,51,79^ have been confirmed by previous studies.

## Conclusion

Our study demonstrates that CT, along with AST supplementation, effectively reduces OS and IF. These improvements are mediated through changes in miRNAs-122, -136-3p and -146a and HN. The observed reductions in OS and IF are associated with favorable alterations in lipid profiles, enhanced IR, and reductions in HbA1c and FBG. Importantly, the combined approach of exercise training and AST supplementation often yields superior benefits compared to each intervention alone, suggesting a synergistic effect that enhances overall metabolic health.

## Limitations

This study only enrolled female participants to minimize inter-individual biological variability arising from sex differences in hormone regulation, fat distribution, and gene expression related to inflammation and oxidative stress. 

## Supplementary Information

Supplementary Information.

## Data Availability

The datasets generated and/or analyzed during this study, including the codebook and analytic code, will be made available to qualified researchers upon reasonable request to the corresponding author(s).
