# Supplementary material for: Redox-sensitive miRNAs and Humanin could mediate effects of exercise and astaxanthin on oxidative stress and inflammation in type 2 diabetes
Source: Sci Rep. 2025 Nov 17;15:40113. doi: 10.1038/s41598-025-23914-y (PMC12623493; doi:10.1038/s41598-025-23914-y)
Supplement: Supplementary file 1 — Supplementary Information. [file 41598_2025_23914_MOESM1_ESM.docx]

**Supplementary**

| ***Table S1. The actual values (Mean ± SD) for all measured variables*** | | | | | |
| --- | --- | --- | --- | --- | --- |
| **Variables** | **Time** | **Pre-test** | | **Post-test** | |
|  | **Group** | **Mean** | **SD** | **Mean** | **SD** |
| **miRNA-122** | **C** | 8.90 | 1.03 | 9.01 | 0.91 |
|  | **P** | 9.40 | 0.72 | 9.48 | 0.67 |
|  | **CT+P** | 9.22 | 0.77 | 6.31 | 0.69 |
|  | **CT** | 8.98 | 0.37 | 6.62 | 0.65 |
|  | **S** | 9.06 | 0.53 | 7.21 | 0.65 |
|  | **CT+S** | 8.91 | 0.60 | 5.02 | 1.08 |
| **miRNA-126-3p** | **C** | 0.09 | 0.02 | 0.08 | 0.02 |
|  | **P** | 0.08 | 0.02 | 0.07 | 0.02 |
|  | **CT+P** | 0.09 | 0.02 | 0.24 | 0.10 |
|  | **CT** | 0.09 | 0.02 | 0.26 | 0.10 |
|  | **S** | 0.09 | 0.02 | 0.18 | 0.05 |
|  | **CT+S** | 0.09 | 0.03 | 0.34 | 0.11 |
| **miRNA-146a** | **C** | 0.11 | 0.03 | 0.11 | 0.03 |
|  | **P** | 0.12 | 0.03 | 0.12 | 0.03 |
|  | **CT+P** | 0.11 | 0.02 | 0.49 | 0.18 |
|  | **CT** | 0.11 | 0.02 | 0.50 | 0.15 |
|  | **S** | 0.10 | 0.02 | 0.39 | 0.16 |
|  | **CT+S** | 0.10 | 0.02 | 0.62 | 0.20 |
| **FBG** | **C** | 136.42 | 5.93 | 136.33 | 4.62 |
|  | **P** | 140.00 | 7.57 | 141.33 | 7.73 |
|  | **CT+P** | 140.67 | 6.50 | 118.25 | 2.63 |
|  | **CT** | 141.42 | 6.93 | 118.08 | 2.91 |
|  | **S** | 142.00 | 7.97 | 134.83 | 9.21 |
|  | **CT+S** | 141.75 | 6.98 | 119.33 | 3.50 |
| **Fasting insulin** | **C** | 12.21 | 0.46 | 12.19 | 0.34 |
|  | **P** | 12.11 | 0.53 | 12.05 | 0.44 |
|  | **CT+P** | 12.15 | 0.39 | 11.60 | 0.48 |
|  | **CT** | 12.22 | 0.47 | 11.52 | 0.37 |
|  | **S** | 12.04 | 0.55 | 11.78 | 0.48 |
|  | **CT+S** | 12.19 | 0.49 | 11.64 | 0.48 |
| **HOMAIR** | **C** | 4.17 | 0.22 | 4.20 | 0.18 |
|  | **P** | 4.18 | 0.20 | 4.24 | 0.21 |
|  | **CT+P** | 4.23 | 0.21 | 3.68 | 0.15 |
|  | **CT** | 4.24 | 0.20 | 3.68 | 0.21 |
|  | **S** | 4.10 | 0.20 | 4.00 | 0.16 |
|  | **CT+S** | 4.18 | 0.21 | 3.62 | 0.33 |
| **Humanin** | **C** | 124.50 | 14.05 | 119.75 | 14.46 |
|  | **P** | 125.75 | 13.82 | 120.33 | 14.18 |
|  | **CT+P** | 125.00 | 9.03 | 151.08 | 17.67 |
|  | **CT** | 127.92 | 6.74 | 154.58 | 19.24 |
|  | **S** | 133.83 | 9.39 | 149.50 | 12.12 |
|  | **CT+S** | 130.75 | 10.25 | 161.83 | 10.32 |
| **GP_X_** | **C** | 9.87 | 1.05 | 9.24 | 1.12 |
|  | **P** | 9.67 | 1.19 | 8.81 | 1.17 |
|  | **CT+P** | 10.07 | 2.10 | 14.26 | 2.43 |
|  | **CT** | 10.58 | 2.12 | 14.77 | 1.98 |
|  | **S** | 9.92 | 1.94 | 12.01 | 1.90 |
|  | **CT+S** | 10.49 | 1.43 | 16.08 | 1.72 |
| **SOD** | **C** | 1.62 | 0.22 | 1.57 | 0.18 |
|  | **P** | 1.48 | 0.27 | 1.44 | 0.25 |
|  | **CT+P** | 1.59 | 0.17 | 1.89 | 0.21 |
|  | **CT** | 1.49 | 0.21 | 1.89 | 0.42 |
|  | **S** | 1.62 | 0.18 | 1.85 | 0.35 |
|  | **CT+S** | 1.69 | 0.26 | 2.20 | 0.32 |
| **IL-36α,** | **C** | 188.67 | 43.92 | 191.33 | 42.05 |
|  | **P** | 191.67 | 52.03 | 201.83 | 52.70 |
|  | **CT+P** | 169.33 | 50.16 | 131.58 | 32.76 |
|  | **CT** | 191.08 | 30.36 | 133.75 | 32.38 |
|  | **S** | 206.67 | 40.22 | 154.92 | 28.44 |
|  | **CT+S** | 201.08 | 46.02 | 118.75 | 26.12 |
| **IL-36γ** | **C** | 406.50 | 124.57 | 408.75 | 97.35 |
|  | **P** | 425.75 | 105.68 | 427.42 | 105.35 |
|  | **CT+P** | 456.00 | 78.78 | 268.42 | 62.47 |
|  | **CT** | 426.42 | 100.71 | 255.58 | 59.74 |
|  | **S** | 364.17 | 95.25 | 294.08 | 80.49 |
|  | **CT+S** | 364.17 | 100.66 | 166.75 | 57.83 |
| **IL-36Ra** | **C** | 94.33 | 31.54 | 90.08 | 31.52 |
|  | **P** | 103.67 | 31.06 | 91.92 | 26.55 |
|  | **CT+P** | 105.00 | 34.63 | 137.92 | 20.62 |
|  | **CT** | 110.08 | 26.16 | 134.58 | 20.46 |
|  | **S** | 83.58 | 32.82 | 121.58 | 26.58 |
|  | **CT+S** | 100.92 | 44.91 | 154.92 | 21.34 |
| **IL17** | **C** | 8.03 | 1.41 | 8.54 | 1.53 |
|  | **P** | 8.28 | 1.47 | 8.54 | 1.52 |
|  | **CT+P** | 8.02 | 1.60 | 6.25 | 1.19 |
|  | **CT** | 8.05 | 1.83 | 5.97 | 1.64 |
|  | **S** | 8.36 | 1.58 | 7.29 | 1.26 |
|  | **CT+S** | 8.55 | 1.31 | 5.82 | 1.30 |
| **TG** | **C** | 255.00 | 20.13 | 262.67 | 25.60 |
|  | **P** | 259.67 | 24.48 | 262.92 | 17.63 |
|  | **CT+P** | 242.50 | 21.65 | 196.08 | 18.30 |
|  | **CT** | 253.17 | 18.30 | 199.75 | 24.63 |
|  | **S** | 258.08 | 21.10 | 232.17 | 24.80 |
|  | **CT+S** | 257.83 | 20.34 | 196.58 | 25.79 |
| **HDL** | **C** | 32.92 | 5.42 | 31.68 | 4.68 |
|  | **P** | 30.67 | 3.77 | 29.92 | 3.03 |
|  | **CT+P** | 30.88 | 4.16 | 36.49 | 4.19 |
|  | **CT** | 30.22 | 4.46 | 36.25 | 4.43 |
|  | **S** | 32.06 | 5.28 | 34.17 | 4.06 |
|  | **CT+S** | 32.35 | 3.85 | 37.99 | 3.41 |
| **HbA1c** | **C** | 7.28 | 0.29 | 7.30 | 0.26 |
|  | **P** | 7.22 | 0.40 | 7.19 | 0.33 |
|  | **CT+P** | 7.34 | 0.21 | 6.68 | 0.25 |
|  | **CT** | 7.31 | 0.28 | 6.73 | 0.27 |
|  | **S** | 7.20 | 0.35 | 6.99 | 0.29 |
|  | **CT+S** | 7.22 | 0.29 | 6.14 | 0.38 |
| **Cholesterol** | **C** | 200.25 | 14.34 | 206.33 | 15.83 |
|  | **P** | 207.42 | 17.69 | 212.75 | 19.52 |
|  | **CT+P** | 197.83 | 15.77 | 178.42 | 17.98 |
|  | **CT** | 200.58 | 17.10 | 181.50 | 19.06 |
|  | **S** | 205.25 | 20.86 | 192.50 | 15.18 |
|  | **CT+S** | 197.42 | 20.80 | 178.50 | 19.35 |
| **TAC** | **C** | 170.25 | 16.58 | 167.08 | 18.89 |
|  | **P** | 178.75 | 17.62 | 174.42 | 16.94 |
|  | **CT+P** | 178.67 | 9.51 | 245.58 | 21.16 |
|  | **CT** | 174.17 | 14.04 | 240.17 | 32.30 |
|  | **S** | 174.67 | 19.49 | 218.00 | 26.88 |
|  | **AT+AS** | 171.33 | 16.32 | 271.17 | 27.13 |
|  | | | | | |

C: control group, P: placebo, CT+P: combined training + placebo, CT: combined training, S: supplement, and CT+S: combined training + supplement.
